# Higher Cortical Dysfunction Presenting as Visual Symptoms in Neurodegenerative Diseases

**DOI:** 10.3389/fneur.2020.00679

**Published:** 2020-07-31

**Authors:** Yin Liu, Victoria S. Pelak, Gregory van Stavern, Heather E. Moss

**Affiliations:** ^1^Department of Ophthalmology, Stanford University, Palo Alto, CA, United States; ^2^Department of Ophthalmology and Vision Science, UC Davis Eye Center, University of California, Davis, Sacramento, CA, United States; ^3^Departments of Neurology and Ophthalmology, University of Colorado, UCHealth Sue Anschutz-Rodgers Eye Center, Aurora, CO, United States; ^4^Department of Ophthalmology, Washington University, St. Louis, MO, United States; ^5^Department of Neurology and Neurological Sciences, Stanford University, Palo Alto, CA, United States

**Keywords:** higher cortical dysfunction, visual symptoms, neurodegenerative diseases, Alzheimer's disease (AD), posterior cortical atrophy (PCA)

## Abstract

**Introduction:** As the population ages, increasing prevalence of neurodegenerative diseases will have profound implications for the health care system. Recognizing visual symptoms from neurodegenerative diseases can be challenging, especially in the presence of co-existing eye diseases.

**Methods:** A seven-question survey was completed by attendees at the “neurodegenerative diseases in neuro-ophthalmology” symposium during the 2017 North American Neuro-ophthalmology Society annual meeting using a web-based audience response system. Content included demographics, patient prevalence, and perceived barriers.

**Results:** Fifty-five practicing neuro-ophthalmologists (thirty-three ophthalmology-trained, twenty-two neurology-trained) participated in the survey. Twenty (36%) had <5 years of experience, and 19 (32%) had >15 years of experience. Forty-one (75%) reported seeing patients more than five half-day/week. Thirty (55%) reported that at least 1 of 10 or 1 of 20 new patients referred have a prior diagnosis of a neurodegenerative disease. Twenty-one (40%) of the respondents reported attributing visual complaints to higher order effects in at least 25% of patients with a prior diagnosis of neurodegenerative disease vs. five (9%) without a prior diagnosis. For those diagnosed with neurodegenerative disease by the neuro-ophthalmologist, reasons for referral were unknown cause of visual symptom (56%), to confirm diagnosis and/or treat visual complaint due to neurodegeneration (29%), and functional disorder (5%). Perceived barriers to diagnosing visual dysfunction due to neurodegenerative disease included difficulty making a referral to neuropsychologists or behavioral neurologists (73%), lack of time for in-depth assessment (62%), lack of tools to assess visual dysfunction due to neurodegenerative disease (40%), and lack of knowledge about presenting signs and symptoms (31%).

**Conclusion:** Visual symptoms from neurodegenerative disease in patients with and without prior diagnoses of neurodegenerative disease are evaluated by neuro-ophthalmologists. Lack of time, resources, and knowledge are barriers to diagnosis. A larger study is warranted to guide programs to improve diagnosis of visual consequences of neurodegenerative disease.

## Introduction

As the population ages, the increasing prevalence of age-related neurodegenerative diseases will have profound implications for health care systems (1). More than 5.5 million people in the United States have Alzheimer's disease and over 1 million have Parkinson's disease (2). Globally, there are almost 46 million people living with dementia. This number is expected to rise to 131.5 million by 2050 (3). WHO Global Burden of disease data shows that dementia is the second largest contributor for total number of years living with disability in people aged 60 years or older at 13.5% comparing to heart disease (4.0%), stroke (4.4%), and cancer (2.2%) (4).

Visual symptoms can present as early signs of neurodegenerative disease and, therefore, are relevant to both diagnosis of neurodegenerative disease and symptom management. For example, posterior cortical atrophy (PCA) is a neurodegenerative syndrome with prominent cortical visual dysfunction with early relative sparing of memory and other cognitive domains that is most commonly associated with Alzheimer's disease (AD) pathology (5).

In this issue, Olds et al. describe the largest retrospective dataset regarding symptoms and signs in patients with PCA presenting to neuro-ophthalmology (6). Data were collected using a retrospective chart review to characterize each patient's history, examination, evaluation, and treatment. Data were also compared to previously published PCA cohorts and on previously published expert opinion regarding the presentation of PCA by cognitive specialists. From a total of 38 patients from 11 institutions and community practices, the PCA cohort presenting to neuro-ophthalmologists had an older age of presentation (68 years) and more likely to be female (3:1) and report difficulty reading (91%) at presentation compared to published data and surveys. Furthermore, poor performance on color vision testing (88%), stereopsis (86%), and visual field testing (89%) were the most common findings on examination and are less likely to be assessed by the cognitive specialist. The results of this survey highlight the importance of considering PCA in older adults who report difficulty reading, have apparent color vision defect on testing, decreased stereopsis, and a visual field defect without explanation. In addition, this study emphasizes the increasingly important role of the neuro-ophthalmologist in the assessment of visual function in neurodegenerative diseases.

## Survey of Neuro-Ophthalmologists Regarding Neurodegenerative Disease in Their Practice

To further understand the landscape and challenges faced by neuro-ophthalmologists consulting on patients with neurodegenerative disorders, we conducted a survey among the attendees at the “Neurodegenerative Diseases and Neuro-ophthalmology” symposium during the 2018 North American Neuro-Ophthalmology Society annual meeting. Conferences i/o web-based audience response system was used to conduct the survey. All participants agreed to anonymous extraction of their survey responses for this project. This study was approved by the Institutional Review Board of Stanford University. The seven-question survey included provider demographics, patient prevalence, and perceived barriers.

A total of 69 attendees participated in the survey. Among them, 55 were practicing neuro-ophthalmologists and 14 were non-neuro-ophthalmologists or trainees. Responses from 55 practicing neuro-ophthalmologists (33 ophthalmology-trained, 22 neurology-trained) were analyzed (Table 1). Twenty (36%) had <5 years of experience, and 19 (32%) had >15 years of experience. Forty-one (75%) reported seeing patients more than five half days per week.

**Table 1:** Summary of survey data on visual symptoms in neurodegenerative diseases.

	**Total *n* = 55**	**Neurology trained *n* = 22, 40%**	**Ophthalmology trained *n* = 33, 60%**
Years in practice 0–5 6–10 11–15 >15	n (%) 20 (38) 8 (15) 7 (13) 18 (34)	3 (15) 6 (75) 3 (43) 8 (44)	17 (85) 2 (25) 4 (57) 10 (56)
Clinical effort (half days/week spent in direct patient care) 1–2 3–4 5–6 7–8 9–10	4 (7) 9 (17) 12 (22) 21 (39) 8 (15)	3 (75) 2 (22) 7 (58) 7 (33) 3 (37)	1 (25) 7 (78) 5 (42) 14 (67) 5 (63)
Frequency of seeing new patients with a prior diagnosis of neurodegenerative disease 1 in every 5–10 patients 1 in every 11–20 patients 1 in every 21–30 patients 1 in every 31 or more patients	5 (9) 25 (46) 13 (24) 11 (21)	3 (60) 14 (56) 3 (23) 1 (9)	2 (40) 11 (44) 10 (77) 10 (91)
Percentage of patients referred for visual complaints and neurodegenerative diseases diagnosed with higher order visual dysfunction by neuro-ophthalmologists ≤ 25% 26–50% 51–75% >75%	33 (61) 13 (24) 5 (9) 3 (6)	13 (39) 5 (38) 2 (40) 2 (67)	20 (61) 8 (62) 3 (60) 1 (33)
Frequency of suspicion for neurodegenerative diseases as the cause for visual complaints in patients without a prior diagnosis of neurodegenerative disease 1 in every 5–10 patients 1 in every 11–20 patients 1 in every 21–30 patients 1 in every 31 or more patients	5 (9) 5 (9) 14 (26) 29 (55)	2 (40) 3 (60) 6 (43) 10 (34)	3 (60) 2 (40) 8 (57) 19 (66)
For patients diagnosed with neurodegenerative disease by a neuro-ophthalmologist, the most common reasons for referral were Could not find other causes No other appropriate providers For formal diagnosis and treatment Evaluate for functional disorders Other	31 (57) 1 (2) 16 (30) 3 (6) 3 (6)	13 (42) 0 (0) 7 (44) 1 (33) 1 (33)	18 (58) 1 (100) 9 (56) 2 (67) 2 (67)
Barriers to diagnosing visual dysfunction due to neurodegenerative disease Difficulty referring to neuropsychologists Lack of knowledge Lack of time in clinic Lack of tools Other	20 (20) 17 (17) 31 (32) 25 (26) 5 (5)	5 (25) 1 (6) 12 (39) 7 (28) 4 (80)	15 (75) 16 (94) 19 (61) 18 (72) 1 (20)

Thirty (55%) of the respondents reported that at least 5–10% of new patients referred to them had a *prior diagnosis* of a neurodegenerative disease, suggesting that these patients are not uncommon in neuro-ophthalmic practice. Twenty-one (40%) reported attributing visual complaints to higher order effects in more than 25% of patients with a prior diagnosis of neurodegenerative disease. The extrapolated prevalence of higher order visual dysfunction with pre-existing neurodegenerative disorder was 302–6,314/100,000, which would be ~1/3–1/2 of all patients with Alzheimer's disease (10,000 per 100,000) if all patients with neurodegenerative disease were to harbor AD. This is similar to the prevalence reported in a very large AD Neuroimaging Initiative Dataset, where Woodward et al. (7) reported cognitive testing with visuospatial worse than memory by >2 SD in 35% mild cognitive impairment and 50% of AD patients. Our survey data is striking with regards to the prevalence of non-neurodegenerative disease related visual symptoms that reaches >90%, many of which might have proven treatment strategies.

For new patients without a prior diagnosis of neurodegenerative disease, 10 (19%) respondents reported attributing visual complaints to undiagnosed neurodegenerative disease in more than 5% of patients. For these patients diagnosed with neurodegenerative disease by the neuro-ophthalmologist, reasons for referral included unknown cause of visual symptom (56%), to confirm diagnosis and/or treat visual complaint due to neurodegeneration (29%), and functional neurological symptom disorder (5%). These data reinforce the important role of experts who can distinguish neurodegenerative from non-neurodegenerative-related visual symptoms.

In our survey, barriers perceived by neuro-ophthalmologists to diagnosing visual dysfunction due to neurodegenerative disease were multifactorial and included: (1) difficulty making a referral to neuropsychologists or behavioral neurologists (73%), (2) lack of time for in-depth assessment (62%), (3) lack of tools to assess visual dysfunction due to neurodegenerative disease (40%), and (4) lack of knowledge about presenting signs and symptoms (31%). Both neurology-trained and ophthalmology-trained neuro-ophthalmologists perceived lack of time in clinic as a common barrier to diagnosing visual dysfunction due to neurodegenerative disease. More ophthalmology trained neuro-ophthalmologists identified lack of knowledge, difficulty referring to neuropsychologist, and lack of tools in clinic as barriers than neurology-trained neuro-ophthalmologists.

## Discussion

Recognizing visual symptoms from neurodegenerative diseases can be challenging, especially in the presence of co-existent eye conditions. Though our survey is limited by selection bias of those who chose to attend a “Neurogenerative Diseases and Neuro-ophthalmology Symposium” and those who agreed to participate, the data collected highlight the role neuro-ophthalmologists are playing in distinguishing visual symptoms from neuro-degenerative and non-neurodegenerative causes for the purposes of diagnosis and treatment as the population continues to age. They also highlight need for education of referring providers and neuro-ophthalmologists to optimize diagnosis and care. Evaluation of visual function in patients with neurodegenerative disorders can be challenging, but familiarity with the type of visual dysfunction most commonly associated with each disorder will allow for identification, focused assessment, and treatment when possible.

Though currently no disease-modifying treatment exists for most neurodegenerative diseases that lead to dementia, early diagnosis, and prompt intervention for visual dysfunction, be they related to neurodegenerative disease or not, can improve quality of life. The data published by Olds et al. regarding the presentation of Posterior Cortical Atrophy syndrome to neuro-ophthalmologists (6) and our survey of neuro-ophthalmology practice trends related to neurodegenerative disease raise important points regarding how assessment and care might be improved for these patients, as neuro-ophthalmologists are frequently positioned to be the definitive diagnostician for patients presenting with higher order visual dysfunction. A larger study is warranted to further characterize barriers to diagnosis and treatment and develop interventions to decrease the obstacles associated with diagnosis and care, in order to guide patient and caregiver education and support, advocacy, and informed policy making.

## Author Contributions

YL analyzed the data, prepared the first draft, and edited the manuscript. VP, GS, and HM conceptualized the survey, collected the data, and edited the manuscript. All authors contributed to the article and approved the submitted version.

## Conflict of Interest

The authors declare that the research was conducted in the absence of any commercial or financial relationships that could be construed as a potential conflict of interest.
